# Remarks on Vermifuges

**Published:** 1850-08

**Authors:** 


*Remarks on Vermifuges.*—Dr. Cazin, of Boulogne-sur-Mer, having had the opportunity of treating a large number of worm cases, has published the following interesting account of his experience. He states that he has frequently employed the common spigelia, or worm-grass. He administers it in the form of decoction, prepared by boiling two drachms of the herb in a quart of water to one-half. The decoction is then expressed, strained, and flavored with a little lemon-juice and a sufficient quantity of sugar. The dose for an adult is two wineglassfuls, followed by a wineglassful every six hours until the desired effect is produced. To children and delicate persons a smaller quantity is to be given.

Wormwood (absinthium) is an excellent indigenous anthelmintic; it is also a powerful tonic and stimulant, the use of which, continued after the expulsion of the worms, prevents their reproduction. M. Cazin often uses a wine prepared by digesting an ounce of wormwood, with an equal quantity of garlic, in a bottle of white wine, of which he gives from one to three ounces every morning. This wine is well-adapted for poor lymphatic subjects, wasted by wretchedness, and suffering from the influence of a marshy soil. The absinthium maritimum is likewise a very good anthelmintic. M. Cazin gives it to the extent of one or two drachms boiled in four or five ounces of water, with the addition of some white sugar, or of any anthelmintic syrup. This is quite a popular remedy in the maritime districts, and almost always succeeds with children affected with worms.

Although a case of poisoning by Cevadilla has been reported, M. Cazin has administered this vermifuge with success in cases in which ordinary anthelmintics had but litte effect; but he has always commenced with a very small dose, in order to ascertain how far it would be borne by the digestive organs. For children, the dose of this plant is from a grain and a half to four or five grains of the powder of the seeds, mixed with syrup of rhubarb; for adults eight or nine grains, with the addition of a little sugar and a few drops of oil of fennel. In each case the dose is to be repeated daily for four days, after which the infusion of chamomile is to be given.

Assafœtida possesses acknowledged anthelmintic properties, and is suitable for cases of sympathetic nervous affections produced by the existence of worms. It thus, like valerian, fulfils a twofold indication. In a case of nervous affection, which M. Cazin believed to be idiopathic, the administration of assafœtida both determined the disease and revealed its true cause, by effecting the expulsion of a number lumbrici. This result has, in three cases of chorea and in two of epilepsy, enabled him to recognize that sympathetic irritation—depending on the presence of intestinal worms—was the sole cause of disease in these instances. Under ordinary circumstances, M. Cazin frequently combines assafœtida with calomel in pills. This combination, of all those that he has employed, succeeds best in expelling lumbrici. He has also combined it with black oxide of iron, particularly in anemic patients. Assafœtida may be given in powder, in doses of from four grains to half a drachm.

The essential oil of turpentine is not merely useful in cases of tenia, it is also decidedly efficacious in expelling the lumbrici. M. Cazin has sometimes, in cases of lumbrici and ascarides, administered with advantage turpentine, enemata, prepared by suspending, by means of yolk of egg, from one drachm to half an ounce of the oil in decoction of tansy, absinthium, worm-seed (semen-contra, or Corsican moss.

Common salt is very destructive to worms; it is given alone in large doses dissolved in water; it should be taken on an empty stomach. M. Cazin also frequently administers it in the form of enema, with brown sugar, linseed or poppy oil, and a sufficient quantity of water. With children it almost always succeeds.

Like all tonics, iron has the advantage of destroying worms, at the same time that, by imparting tone to the intestines, it prevents their reproduction. From six to eight grains of iron filings, mixed with an equal quantity of rhubarb, and taken twice or three times a day, have often been sufficient to expel the worms contained in the intestines. M. Cazin succeeded in rapidly curing a boy nine years of age, emaciated and pale, whose sleep was disturbed, and who was suffering from spasmodic movements similar to those which characterize chorea, by the exhibition of pills of sulphate of iron, combined, according to Fuller's formula, with aloes, senna, &c., under which treatment he voided twenty-three lumbrici in four days. He has also used with remarkable success Bosen's mixture, containing extract of black hellebore and sulphate of iron. But what he chiefly gives to children, as well as to adults, is the syrup of citrate of iron (four parts of citrate to sixty of simple syrup, and one of essence of lemon,) in doses of from two drachms to half an ounce to children, and from half an ounce to two ounces to adults.

M. Cazin remarks that calomel, so efficacious as an anthelmintic, ought never to be combined with an alkaline chloride, as the formation of corrosive sublimate would probably ensue from their admixture. In like manner, the combination of calomel with cherry-laurel water, or emulsion of bitter almonds, would give rise to the development of two formidable poisons, corrosive sublimate and cyanide of mercury.

The effects of the male fern, tin, pomegranate bark, hellebore, &c., require merely to be noticed; and the properties of the pomegranate root bark are so well known that they need not be dwelt upon. M. Cazin has remarked nothing particular respecting other anthelmintics. He merely says that cod-liver oil has succeeded with him in the cases of two females, one of whom passed twelve lumbrici the same day that she had taken in the morning three tablespoonfuls at intervals of an hour.

But, whatever be the medicine selected, we must not, like routine practitioners, be content, when the worms are killed and dislodged, with this merely palliative cure. A very important indication remains to be fulfilled, viz.: to prevent their reproduction. This object is attained, according to M. Cazin, by the adoption of a tonic and stimulant regimen, which must be long continued, and, above all, by the employment of bitter and chalybeate preparations. He has found the ferruginous chocolate to be sufficient, in the case of children, to prevent the relapses which are for many years very apt to occur. Wine taken while fasting has succeeded with the poor inhabitants of the marshes, accustomed to live only on vegetables and milk; and he has also remarked its efficacy as a preventative of worm affections in other instances.

To these observations of M. Cazin, the editor of the Journal de Médecine has appended the following practical remarks. The number of experiments tried by M. Cazin leaves no room for doubt respecting the enormous amount of worm affections which he must have met with. Such a result may appear strange to Parisian physicians, who attribute to the presence of worms in the intestines only a very trifling influence over the symptoms formerly ascribed to them. But if worm affections are rare among the inhabitants of large towns, they are frequent and generally more serious among the peasantry, and particularly among those who are poor and placed in unfavorable hygienic circumstance. We shall, therefore, take the present opportunity of mentioning the efficacy of *brown santonine*, lately brought under the notice of the readers of the Bulletin de Thérapeutique, by M. Gaffard, an apothecary at Aurillac.

The difficulty experienced in procuring pure santonine, both on account of its high price, and for other reasons, has induced M. Gaffard to endeavor to obtain from *worm-seed*, a product which may possess the advantages of the former, and at the same time be free from the objections to the use of the latter. This product he calls brown or impure santonine; it is obtained in the following manner:

Take of Aleppo worm-seed, three ounces; carbonate of potash, one ounce; slacked lime, sifted, half an ounce; water, from three pints to three pints and a half. Place the mixture on the fire, stirring occasionally with a wooded spatula; let it boil for an hour; on removing it from the fire pass it with expression through a linen cloth, let it settle, decant, and add hydrochloric or nitric acid until it reddens litmus without being sensibly acid to the tongue. Allow it to rest, pass it through a filter previously moistened, or through a piece of close canvas, and allow the product which remains on the filter to dry in the open air until it acquires the consistence of firm butter. This product, which is a mixture of santonine, resin, and essential oil, will answer for the various pharmaceutic forms in which the practitioner may wish to exhibit it. M. Gaffard gives it in the form of lozenges composed as follows:

Brown santonine, three drachms; powdered sugar, thirteen ounces; powdered gum, one ounce and a half; essential oil of lemon, twenty-five drops. Place the brown santonine in a marble mortar; add by degrees, and with constant trituration, the sugar mixed with the essential oil and the gum, so as to make a homogenous powder. Form with a sufficient quantity of water a mass of the desired consistence, and divide it into lozenges, each of which shall weigh, when dried, fifteen grains; each lozenge will then contain somewhat more than one-third of a grain of brown santonine.

For infants under six months the dose will be one lozenge night and morning; from six months to a year, two lozenges night and morning; from one to two years, three; and from two to four years, four night and morning; for children of five years and upwards a lozenge for each year of the child's age should be given night and morning. The medicine to be continued until the desired effects are produced.—*Journal de Médicine et de Chirurgie Pratiques*, March, 1850.

